# “The further along I get the more I feel like growth is possible”: lived-experience narratives of the shape of recovery in borderline personality disorder

**DOI:** 10.64898/2026.10.08.26365022

**Published:** 2026-10-11

**Authors:** Verenice Torres, Thea M. McAfee, Paulina Tein, Alexandra A. Alario, Wenjing Luo, Kaylin Foss, Sarah K. Fineberg, Sharon M. Nelson

## Abstract

Borderline personality disorder (BPD) has traditionally been viewed as one of the most chronic psychiatric disorders. Although longitudinal studies have demonstrated promising rates of symptomatic remission, recovery and other clinical outcome measures often remain comparatively low. Qualitative research offers an important perspective on how individuals with BPD may extend a more representative view of improvement and change. We collected narratives from 41 participants with a self-reported diagnosis of BPD and analyzed them using thematic analysis. Its national, virtual recruitment approach enabled a relatively large qualitative sample diverse in gender identity, age, U.S. geographic region, treatment history, and current clinical severity, extending the range of perspectives represented in prior work. Three central themes were identified. The first, *Differing attitudes toward language* , reflected participants’ experiences of recovery language as paradoxically both validating and stigmatizing. While individual language preferences varied, participants generally converged on a shared understanding of what recovery meant to them. The second, *Recovery as a dynamic and relational process* , described recovery as nonlinear, effortful, and closely tied to interpersonal experiences. The third, *Crossroads and uneven change over time* , highlighted the role of personal agency, critical decision points in seeking treatment, and the persistence of internal distress even as behavioral symptoms improve. Findings from this study both replicate and extend existing qualitative research by discussing how individuals with BPD understand recovery in ways that may not be captured by traditional clinical outcome measures. These results emphasize the importance of incorporating lived-experience perspectives when defining recovery and setting treatment goals.

## Full Text Availability

The license terms selected by the author(s) for this preprint version do not permit archiving in PMC. The full text is available from the preprint server.

